# An Intestinal Intravascular Large B-Cell Lymphoma Presenting as Small Bowel Ischemia: A Case Report and Literature Review

**DOI:** 10.7759/cureus.100447

**Published:** 2025-12-30

**Authors:** Nobuko Matsuoka, Yoshihito Kakimoto, Hideto Shiraki, Shuhei Ota, Teruyoshi Amagai

**Affiliations:** 1 Department of Surgery, Yao Tokushukai General Hospital, Osaka, JPN; 2 Faculty of Health Care Sciences, Department of Clinical Engineering, Jikei University of Health Care Sciences, Osaka, JPN

**Keywords:** explanatory laparoscopy, general emergency surgery, intestinal ivlbcl, intravascular large b-cell lymphoma (ivlbcl), small bowel ischemia

## Abstract

Intravascular large B-cell lymphoma (IVLBCL) is a rare type of malignant lymphoma characterized by the proliferation of neoplastic cells within small blood vessels. Here, we present a rare case of small intestinal IVLBCL presenting with intestinal necrosis. A 78-year-old man presented with vomiting and abdominal pain. Contrast-enhanced CT revealed an edematous, poorly enhanced small intestine, suggestive of ischemia. Emergency laparoscopy was performed based on radiological findings indicating intestinal ischemia, and a 50 cm segment of necrotic ileum was identified. This segment was resected with adequate margins and reconstructed using an end-to-end anastomosis. Embolic material was noted in the mesenteric vessels. Histopathological examination revealed large, uniform lymphoid cells within vascular lumina and lymph node sinuses. Additional immunohistochemical analysis showed positivity for CD20 and Ki-67 (80-90%), consistent with high-grade IVLBCL. The patient was transferred to the hematology department to initiate chemotherapy with the R-CHOP regimen. IVLBCL often lacks typical physical features, such as lymphadenopathy or mass formation, making diagnosis difficult. Intestinal IVLBCL, a rare malignant lymphoma characterized by the selective proliferation of tumor cells within the lumen of small- to medium-sized blood vessels, represents a GI manifestation of IVLBCL. This case report describes a 78-year-old man who presented with vomiting and abdominal pain and was diagnosed with IVLBCL.

## Introduction

Intravascular large B-cell lymphoma (IVLBCL) was first described in 1959 by Pfleger and Tappeiner as a very rare form of diffuse large B-cell lymphoma that proliferates within small blood vessels [1]. According to the 2008 WHO classification, it is categorized as a rare type of non-Hodgkin lymphoma [2]. It is defined as a disease in which large B cells are present primarily within blood vessels, without lymphadenopathy or obvious tumor formation. Historically, when first reported, it was considered an angioendothelial tumor. However, since the 1980s, immunohistochemical studies using monoclonal antibodies (CD20, CD79a, PAX5, etc.) have demonstrated that it is a B-cell tumor, leading to its classification as a B-cell lymphoma [3]. The term “large cell” refers to the tumor cells being larger than normal lymphocytes, distinguishing this entity from small-cell and indolent lymphomas. Therefore, the designation IVLBCL reflects both the intravascular localization and large B-cell morphology, making it pathologically and taxonomically appropriate. This definition has been maintained in recent classifications, including the latest WHO/HAEM5.

Review articles report the most common age range at onset to be 34-90 years, with a median age of 60-70 years [4]. The current WHO classification describes three subtypes of IVLBCL: the classic subtype, cutaneous subtype, and hemophagocytic subtype [5]. Neurological symptoms and disease restricted to the skin (i.e., the cutaneous subtype or so-called classical type) are more frequently observed in patients from European countries [6]. In contrast, the hemophagocytic subtype is considered more prevalent in Asian countries, including Japan. In 2014, Fonkem et al. retrospectively analyzed 740 cases of intravascular lymphoma reported between 1959 and 2011, of which 651 were IVLBCL [7]. This study found involvement of the CNS, bone marrow, spleen, skin, and lung in 60%, 11%, 11%, 8%, and 7% of cases, respectively. The frequency of organ-specific involvement varies, with higher prevalence reported in the CNS, skin, and endocrine organs, including the thyroid and adrenal glands [8].

The clinical presentation of IVLBCL ranges from monosymptomatic or paucisymptomatic forms, such as fever of unknown origin, pain, or organ-specific local symptoms, to the presence of B symptoms and signs of multiorgan failure. However, reports of primary IVLBCL involving abdominal organs, particularly the digestive tract, are relatively rare. Among 30 patients with an in vivo diagnosis of IVLBCL, only three cases involved the intestinal tract [9]. This case report presents a case of primary IVLBCL of the small intestine, along with a review of ten reported abdominal IVLBCL cases and a literature review.

## Case presentation

A 78-year-old man with a medical history of hypertension and otherwise in good health presented to the emergency department with complaints of vomiting and abdominal pain. He had experienced abdominal discomfort and distension since the previous day, and vomiting developed on the day of admission, prompting an emergency call. He was independent in activities of daily living. His height was 165 cm, weight was 55.8 kg, and body mass index was 20.5 kg/m². He had quit smoking 15 years earlier and reported occasional alcohol consumption.

On arrival, he was alert and fully oriented, with a Glasgow Coma Scale score of 15 points (M6V5E4). In fully alert patients, the motor response (M), verbal response (V), and eye-opening (E) components score 6, 5, and 4 points, respectively [10]. His vital signs were largely within normal limits: blood pressure, 140/58 mmHg; heart rate, 58 bpm; and body temperature, 35.4°C. No superficial lymphadenopathy was noted. Abdominal examination revealed marked distension, diffuse tenderness, and spontaneous pain centered in the left lower quadrant. There was no rebound tenderness.

**Figure 1:**
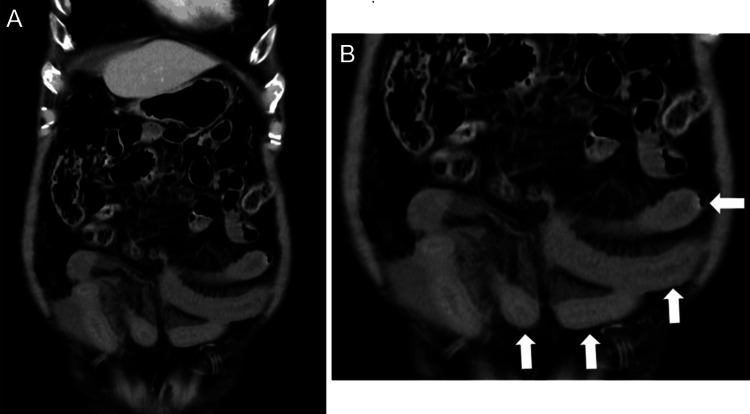
Extensive small intestinal ischemia suspected on contrast-enhanced CT (A) Contrast-enhanced abdominal CT scan. (B) Magnitude image showing dilated and edematous segments of the small intestine with poor enhancement (arrows), suggestive of strangulated small bowel obstruction, without evidence of lymph node enlargement.

Laboratory data indicated an elevated C-reactive protein level (2.12 mg/dL; reference range: <0.14 mg/dL), suggesting systemic inflammation. Contrast-enhanced abdominal CT revealed dilated and edematous segments of the small intestine with poor enhancement, suggestive of strangulated small bowel obstruction (Figure 1).

**Figure 2:**
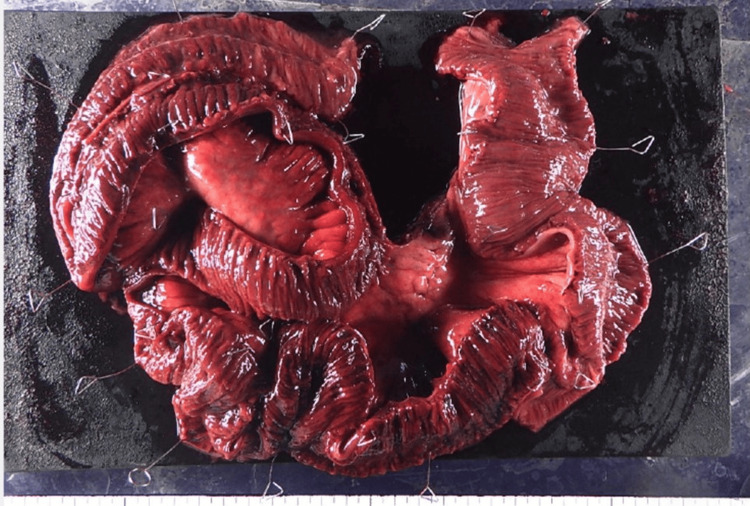
Surgical specimen showing a 50 cm segment of necrotic small intestine removed during emergency laparotomy Examination of the superior mesenteric vessels revealed embolic material within the vascular stump and enlarged lymph nodes, suggesting mesenteric arterial embolism as the cause of bowel necrosis.

Based on these findings, an emergency exploratory laparoscopy was performed for a suspected acute abdomen. Intraoperative findings revealed approximately 50 cm of necrotic small intestine, prompting conversion to an open laparotomy. Examination of the resected mesenteric vessels revealed embolic material within the vascular stump. This finding raised suspicion that mesenteric arterial embolism was the cause of the bowel necrosis (Figure 2).

**Figure 3:**
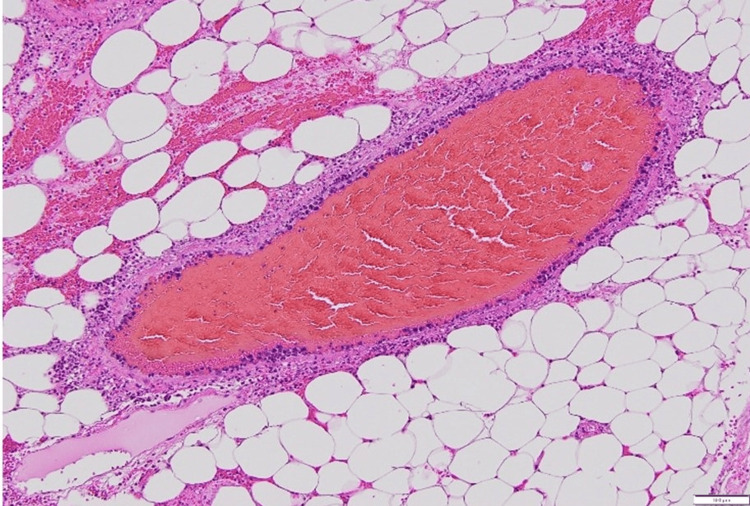
Pathological specimen stained with H&E H&E staining revealed lumina of small- to medium-sized blood vessels filled with large, highly atypical lymphoid cells. There was little evidence of extravascular invasion, and the surrounding adipose tissue and stromal architecture were relatively well preserved. Tumor cells exhibited large nuclei and coarse chromatin.

**Figure 4:**
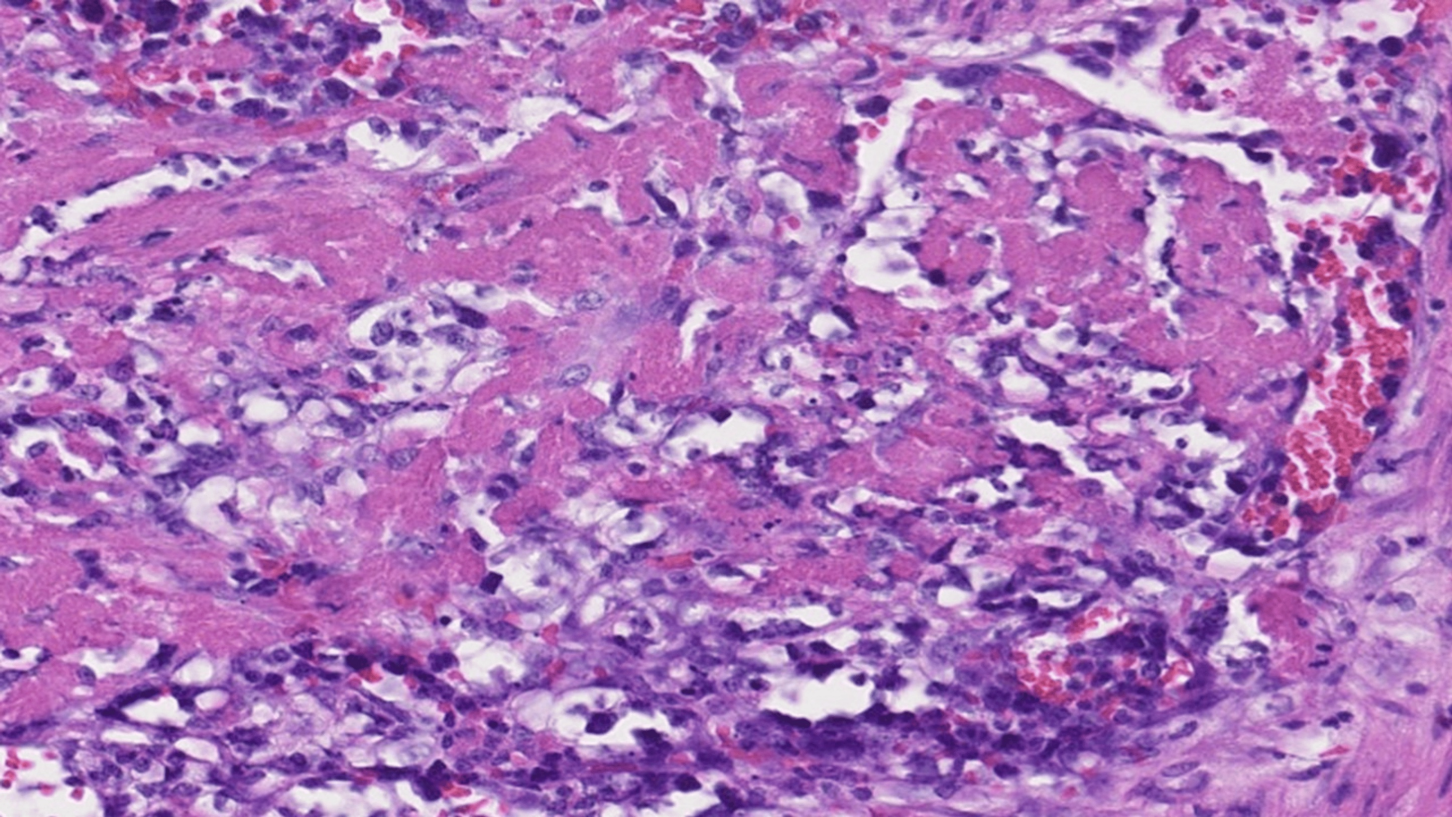
Pathological specimen of lymph nodes stained with H&E H&E staining revealed dense proliferation of large, atypical lymphoid cells within small vessels and capillary spaces. Dilated vascular spaces were filled with tumor cells, with minimal infiltration into the extravascular stroma. Surrounding hemorrhage and inflammatory cell infiltration were present. Tumor cells demonstrated large, round-to-irregular nuclei with marked nuclear atypia.

The necrotic segment of the small intestine was resected, and reconstruction was performed using a functional end-to-end anastomosis. The total operative time was one hour and 25 minutes, with an estimated blood loss of 170 mL. Pathological examination revealed large, relatively uniform lymphoma cells proliferating within the vascular lumina and walls of the small intestinal mesentery, as well as within lymph node sinuses (Figure 3, Figure 4).

Additionally, immunohistochemical staining showed CD20(+), Ki-67 labeling index 80-90%, CD3(−), CD5(−), CD10(−), CD30(−), BCL-6 (− to weak+), MUM1 (− to weak+), and EBER (−). The diagnosis of high-grade IVLBCL was confirmed based on this immunohistochemical profile, which is characteristic of the disease and has been previously reported [11]. Due to the absence of a hematology department at our hospital, the patient was transferred to a specialized facility and initiated on R-CHOP therapy.

## Discussion

Epidemiology

IVLBCL is a rare lymphoma subtype that lacks the typical features of malignant lymphoma, such as lymphadenopathy, extravascular tumor formation, and peripheral blood involvement, making diagnosis challenging. Antemortem diagnosis is achieved in only ~50% of cases, with a median survival of five months [12]. Early diagnosis and prompt treatment are critical. A large, population-based Anglo-American study reported that the age-adjusted incidence rate in the United States between 2000 and 2013 was approximately 0.095 per 1,000,000 person-years, with a median age at diagnosis of 70 years [3]. Other review articles report the typical age range as 34-90 years, with a median age of 60-70 years [4].

Symptomatology

Patients diagnosed with IVLBCL often present with non-specific symptoms, such as fever, malaise, and unintended weight loss, without lymphadenopathy [8]. Intravascular infiltration at the microvascular level in multiple organs, including the kidneys, spleen, skin, and bone marrow, can lead to organ dysfunction, resulting in conditions such as renal impairment or pulmonary lesions.

Diagnosis

Biopsies of organs like the brain, kidneys, and lungs are highly invasive and carry risks for pathological diagnosis of IVLBCL. Therefore, less invasive skin biopsies, termed “random skin biopsies” (RSB), are often used for early diagnosis [12]. RSB should be considered in patients with clinical features such as fever of unknown origin, elevated LDH levels, absence of lymphadenopathy, and non-specific blood findings, including cytopenia and skin rash [13]. Care is required when performing RSB from subcutaneous fat-rich areas such as the thigh, abdomen, or posterior upper arm [14], due to the risk of bleeding or hemorrhagic shock in patients with pancytopenia. Punch biopsies may also yield false negatives because they collect insufficient adipose tissue [15].

A recently proposed IVLBCL probability scoring system may also aid diagnosis [16]. This system uses three laboratory values: serum LDH < 256 IU/L, serum sIL-2R < 2,011 U/mL, and platelet count > 107 × 10⁹/L. Patients with scores < 2 were not diagnosed with IVLBCL, whereas probabilities of IVLBCL in patients with scores of 2 and 3 were 18% and 86%, respectively.

Incidence of primary organ involvement

The most common primary tumor site is the CNS, accounting for 51.6% (94 of 182) of aggregated cases [17]. The bone marrow is the next most frequent site, accounting for 41.0% (73 of 182) in one review and 45.1% (92 of 204) in another review of 331 cases [18]. Other affected organs include the lungs (30.8%, 56 of 182 cases), skin (19.8%, 36 of 182 cases), and endocrine organs, including the adrenal, pituitary, and thyroid glands. The abdomen is a relatively common site for reticuloendothelial system involvement, with reports of hemophagocytic syndrome affecting the liver and spleen [19]. However, thrombus formation in the vessels supplying the digestive tract is rare.

Intestinal IVLBCL and poor prognosis

A 15-year literature search (2010-2025) in PubMed and PMC using the terms “mesenteric vein thrombosis” and “IVLBCL” identified 12 cases (Table 1) [20-31]. A 2016 case report of colonic IVLBCL referenced two previously reported cases in the GI tract [23] and noted that the GI tract is an exceedingly rare primary site for IVLBCL. Among these 12 cases, the mortality rate was 66.7% (8/12). Analysis revealed that cases often had LDH levels exceeding 633 U/L. A retrospective analysis of 1,221 patients between 2003 and 2006 showed that large B-cell lymphoma involving the small intestine, whether intravascular or diffuse-type, is associated with poor overall survival [32]. Based on these observations and our case, IVLBCL originating in the small intestine carries a poor prognosis.

**Table 1:** Clinical profiles of the 12 cases of intestinal IVLBCL reported in the literature A literature search identified 12 cases of intestinal IVLBCL reported since 2010. Patients’ ages ranged from 31 to 85 years, and eight of the 12 patients died, resulting in a mortality rate of 66.7%. ^*^: Headache, dizziness, and facial edema; +: presence of symptom; -: absence of symptom IVLBCL: intravascular large B-cell lymphoma; LDH: lactate dehydrogenase; ND: not determined; WL: weight loss

No.	Age	Sex	Complaint			LDH (U/L)	Prognosis	Follow-up period	Reference
			Abdominal pain	Fever	Others				
1	76	Male	-	+	WL, malaise	1097	Death	3 days	[20]
2	79	Male	+	-	None	702	Death	2 years	[21]
3	61	Female	-	+	Melena, malaise	1233	Death	30 days	[22]
4	47	Female	-	-	Rectal lesion	ND	Alive	12 months	[23]
5	65	Female	-	+	None	ND	Death (autopsy)	ND	[24]
6	31	Female	+	-	Pregnant^*^ (16 weeks)	ND	Alive	18 years	[25]
7	85	Female	-	+	Epigastric pain	633	Death	21 days	[26]
8	69	Male	-	-	None	ND	Death (autopsy)	ND	[27]
9	58	Male	+	-	Pancytopenia	625	Alive	12 months	[28]
10	71	Female	-	-	Hypoxia, hemolytic anemia	ND	Death (autopsy, Case 2)	ND	[29]
11	70	Male	+	-	General feeling of weariness	920	Alive	6 months	[30]
12	75	Male	+	-	ND	ND	Death	7 days	[31]

## Conclusions

Intestinal IVLBCL is a rare malignant lymphoma characterized by the selective proliferation of tumor cells within the lumen of small- to medium-sized blood vessels. It represents a form of IVLBCL presenting in the GI tract. This case report describes a 78-year-old man who presented with vomiting and abdominal pain and was diagnosed with intestinal IVLBCL. He survived for 12 months following surgical resection of the ischemic small intestine and treatment of superior mesenteric vein thrombosis associated with IVLBCL.
